# An ecological assessment of decision-making under risk and ambiguity through the virtual serious game Kalliste Decision Task

**DOI:** 10.1038/s41598-024-63752-y

**Published:** 2024-06-07

**Authors:** Francisco Molins, José-Antonio Gil-Gómez, Miguel Ángel Serrano, Patricia Mesa-Gresa

**Affiliations:** 1 Department of Psychobiology, Universitat de València, Av. Blasco Ibáñez, 13, 46010 Valencia, Spain; 2 Instituto Universitario de Automática e Informática Industrial, Universitat Politècnica de València, Valencia, Spain

**Keywords:** Decision-making, Risk taking, Serious games, Ecological assessment, Loss aversion, Decision, Neuroscience, Psychology

## Abstract

Traditional methods for evaluating decision-making provide valuable insights yet may fall short in capturing the complexity of this cognitive capacity, often providing insufficient for the multifaceted nature of decisions. The Kalliste Decision Task (KDT) is introduced as a comprehensive, ecologically valid tool aimed at bridging this gap, offering a holistic perspective on decision-making. In our study, 81 participants completed KDT alongside established tasks and questionnaires, including the Mixed Gamble Task (MGT), Iowa Gambling Task (IGT), and Stimulating & Instrumental Risk Questionnaire (S&IRQ). They also completed the User Satisfaction Evaluation Questionnaire (USEQ). The results showed excellent usability, with high USEQ scores, highlighting the user-friendliness of KDT. Importantly, KDT outcomes showed significant correlations with classical decision-making variables, shedding light on participants’ risk attitudes (S&IRQ), rule-based decision-making (MGT), and performance in ambiguous contexts (IGT). Moreover, hierarchical clustering analysis of KDT scores categorized participants into three distinct profiles, revealing significant differences between them on classical measures. The findings highlight KDT as a valuable tool for assessing decision-making, addressing limitations of traditional methods, and offering a comprehensive, ecologically valid approach that aligns with the complexity and heterogeneity of real-world decision-making, advancing research and providing insights for understanding and assessing decision-making across multiple domains.

## Introduction

Assessing decision-making capacities is complex due to the diversity of environments in which this cognitive function can occur, as well as the numerous influencing factors to which it may be subject. As an alternative that aims to overcome the limitations of conventional assessments, this study introduces the Kalliste Decision Task (KDT), a virtual environment designed to comprehensively evaluate decision-making. Following an overview of the current state of the art, we will describe KDT, assess its usability, and explore its ability to predict performance in various decision-making settings.

Our life is full of decisions. From the simplest to the most complex choices, most involve some risk, requiring a cost–benefit evaluation. This evaluation depends, in turn, on working memory, attention, sensitivity to feedback, impulse control, and many other cognitive skills that underlie the complex decisional process^1–4^. However, environmental constraints are also important. Depending on the context, different cognitive skills are required to decide^5–7^. Decision contexts are usually classified along a continuum of uncertainty^7,8^. At one extreme would be *risk contexts*, where the decision options or prospects, as well as the probabilities of different outcomes, are well known. In these contexts, it would be possible to follow a rule-based, calculated, rational strategy to maximize the utility of decisions^7^. Conversely, at the end of the continuum would be *ambiguous contexts*, where information is imperfect, prospects and outcomes could be even unknown, and choices should be based on intuition and reinforcement learning from previous experiences^5,7^.

Considering this heterogeneity is necessary to avoid biased conclusions about decision-making capacity. Most studies on decision-making are conducted in risk scenarios and take rationality as the only criterion of evaluation^9,10^. The simplicity of this context allows isolation of the variables of interest, avoiding multiple confounding factors, but may suffer from a lack of ecological validity^11,12^. In clinical population, classical studies carried out by Bechara & Damasio^13^ have already revealed this issue: while some patients with emotional brain lesions can still make rational decisions in risk laboratory settings, they have significant difficulties in learning by reinforcement and making adaptive decisions in complex and ambiguous real-world contexts. Another example is autism spectrum disorder (ASD), where patients tend to make less biased decisions in risk contexts^14,15^ but struggle to adapt in complex decision-making scenarios, especially in social environments^16,17^. Additionally, many heuristics and biases that challenge principles of rationality in risk contexts may actually be adaptive in other settings^12,18,19^. For instance, the loss aversion bias, in which losses are more important than gains^20^, underlies various irrational consumer behaviors such as the framing effect, endowment effect, and the *status quo* bias^21^. However, recent research suggests that loss aversion may also play a protective role against self-injurious behaviors^22^ and even suicide attempts^23,24^.

For these reasons, new lines in decision-making research argue for the need to not only judge decisions based on rationality in risk contexts, but to consider multiple criteria in interaction with several contexts^12^. Indeed, it has even been proposed to evaluate the decision-making process and not just the consequence, as many outcomes may be the result of uncontrollable factors^25^. However, these approaches are not yet common and there is no standardized way of applying them.

Some authors directly compare decisional performance between risk and ambiguous contexts^26^, being the mixed gamble task (MGT)^27^ and the Iowa gambling task (IGT)^28,29^ the most prominent ways to study, respectively, risk and ambiguous decisions. However, in these tasks, subjects are confronted with controlled stimuli and previous results show that, in isolation, they have a weak correspondence with real-life decisions^30–32^. In contrast, another research opts for addressing throughout self-reports a wide range of psychological constructs that can influence decision-making performance, such as risk attitude/propensity, personality traits or impulsivity^33–35^. But again, matching isolated self-report measures to real-world decisions may lead to low-validity conclusions^36^. Moreover, there is the “ask or task” debate, which states that self-reports could provide measures closer to traits, while tasks would capture more situational measures^37^. Therefore, it would be necessary to combine previous approaches to comprehensively assess decision-making and be able to extract firm conclusions. However, an extensive measurement may lead to participants fatigue and lack of motivation during the evaluation, also impairing ecological validity.

Fortunately, and due to recent technological advances, Serious Games (SG) have emerged as a powerful, engaging, and adaptive tool that can overcome the limitations of conventional methods^36,38^. Virtual environments can be constructed to emulate a wide range of decisional contexts, from risk to ambiguous scenarios, being able to analyze how people face them and which outcomes they achieve. This evaluation constitutes a stealth assessment^36^: an unnoticed and continuous real-time measurement, which can assess not only explicit decisions, but also implicit or embodied-decisions (i.e., more natural decisions that are not consciously filtered). Previous evidence on SG has shown significant relationships between the neural mechanisms that subjects experience when immersed in virtual environments and in real life^39–42^. Indeed, recent SG addressing risk-taking, such as the Spheres & Shields Maze Task^43^, can significantly predict risk-related factors such as impulsivity, extraversion, or hazardous behaviors like marijuana consumption. Nevertheless, although there are several SG that address risk-taking (an important component of the decisional process), to our knowledge, there is no SG that comprehensively addresses decision-making.

In this study, we introduce Kalliste Decision Task (KDT) as a virtual environment for a comprehensive and ecological decision-making assessment. KDT is a SG that sets the goal of maximizing monetary gains within a defined time limit. To achieve this, explicit and implicit decisions must be made throughout multiple rooms and corridors. As will be described in depth in the Methods section, through interaction with various tokens (e.g., coins or boxes), KDT presents a heterogeneous decisional environment, with decision-making ranging from complete uncertainty (ambiguous contexts^5,7^) to reduced uncertainty where it is indicated the possible outcomes and their probabilities when deciding (risk contexts^7^). Additionally, the level of danger, which primarily depends on the number of enemies and traps, varies throughout the environment, facilitating an analysis of the extent of risk undertaken. This heterogeneous setting allows for a comprehensive assessment of each participant’s decision-making processes. The objectives of this research are to widely describe KDT, evaluate the usability of this software, and validate it with healthy subjects. The main aim is to demonstrate its effectiveness as an assessment tool in the field of decision-making. The specific research hypotheses are delineated as follows: the game was designed to emulate the settings of other serious games similar to the KDT framework^43,44^, with the intention of creating an immersive, engaging, and user-friendly experience. Consequently, following its administration, (H1) participants will report elevated scores on the User Satisfaction Evaluation Questionnaire (USEQ)^45^. On the other hand, we expect that exploratory analyses will reveal significant correlations between behaviors displayed in KDT and traditional decision-making metrics. More specifically, considering that interaction with KDT tokens involves a range of decisional contexts (see Methods section for details), it is hypothesized that (H2) outcomes derived from risk contexts will be particularly correlated with scores from the MGT^27^; those derived from ambiguous contexts will be primarily correlated with scores from the IGT^28,29^; and finally, risk-taking behaviors will be associated with self-reported levels of instrumental and stimulating risk-taking. Complementarily, and in line with the pursuit of more advanced and precise models that comprehensively address decision-making^46–48^, we conducted hierarchical cluster analyses. It is expected that these will classify participants into well-differentiated decisional profiles based on behavioral similarities observed within the video game environment (H3). Finally, (H4) these delineated profiles are expected to exhibit significant differences in the aforementioned classical decision-making metrics.

## Methods

### Participants

Previous studies have validated closely related tools, such as the Spheres & Shields Maze Task^43^ or the Assessment on Decision Making in Risk Environments (AEMIN) tool^44^. Although these studies did not report effect sizes that allow for the estimation of the required sample size, they obtained promising results with samples of 41 and 98 participants, respectively. Based on the sample size of these studies, an average of 70 participants is estimated. However, we oversampled by an additional 13 participants to ensure the acquisition of results, preventing potential issues that could reduce the final sample. 83 individuals were recruited to participate in the study. Yet, two participants did not complete the experiment, so our sample was finally composed by a total of 81 participants (age: *M* = 19.73, *SD* = 2.72; women: *N* = 52, 64.2%). All of them, students from the University of Valencia, were recruited in the classes by asking them if they wanted to voluntary participate. They met the following inclusion criteria: not having neurological, or psychiatric diseases; not consuming more than 5 cigarettes a day; not consuming drugs habitually and not having experienced a highly stressful event in the last month.

### Kalliste decision task (KDT)

**Figure 1:**
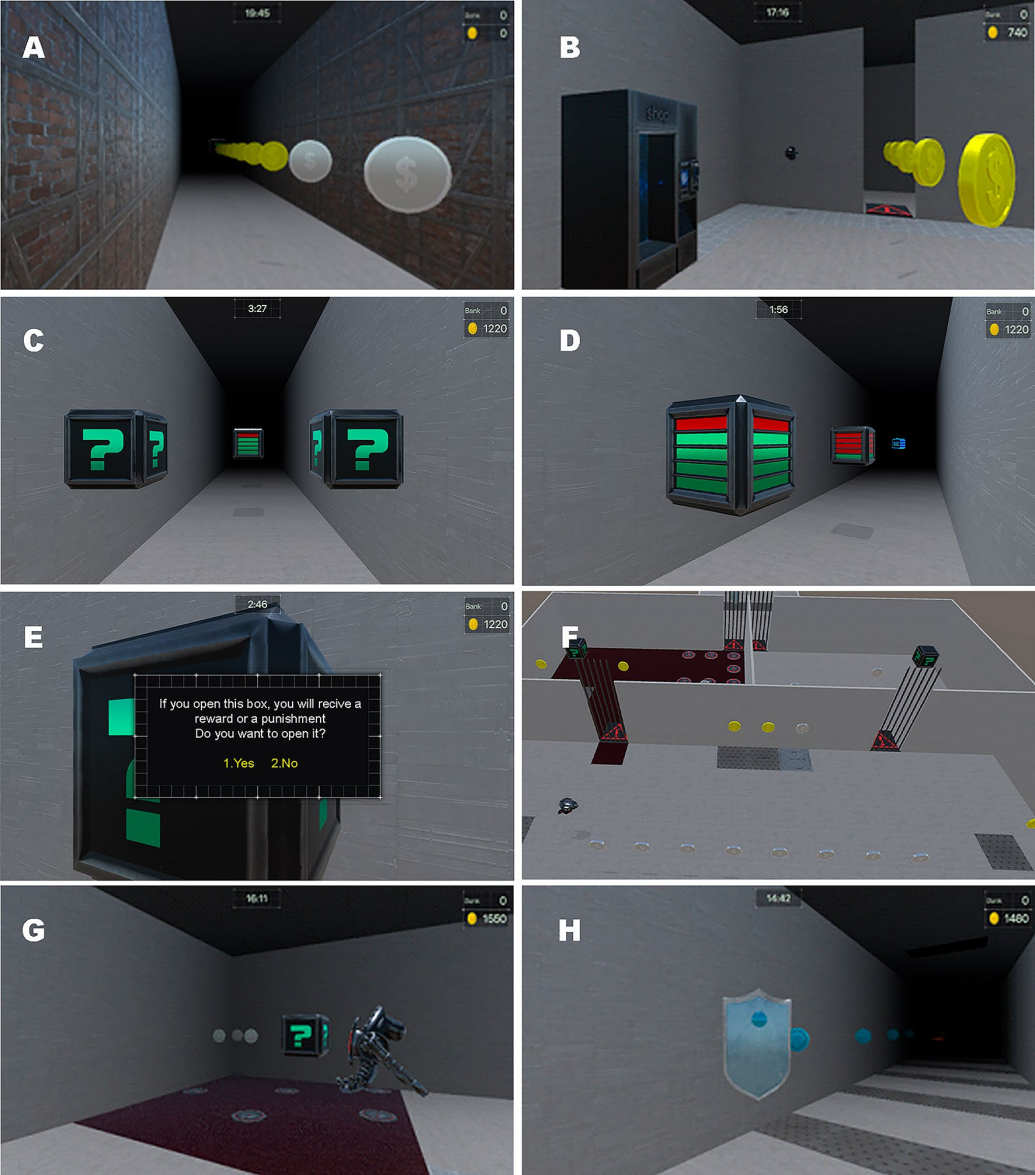
The Kalliste Decision Task (KDT) environment. Participants must earn as much money as possible before time runs out. They can do this by (**A**) collecting coins throughout the stage and (**B**) investing in vending machines to buy extra time. In addition to coins, participants can increase their funds by opening boxes. There are (**C**) ambiguous boxes marked with a question mark, offering no clues about the chances or the amount at stake. There are also (**D**) risk boxes, with varying probabilities of winning (greener for higher chances, redder for lower) and clear indicators of potential gains or losses. Upon approaching any box, (**E**) participants will be asked if they wish to open it. (**F**) The stage also features multiple forks, leading to similar halls or rooms. Those with red floors are more dangerous, promising greater risk and guaranteed losses. For instance, (**G**) a robot patrols around a box, surrounded by mines, which, if opened, results in financial loss. Conversely, (**H**) grey floors pose less danger, and any threats can be easily avoided, such as by using shields.

KDT is an interactive virtual environment designed to comprehensively assess the participants’ decision-making (see to Fig. 1 for an overview of KDT). From the outset, the aim of KDT is clear: participants should strive to maximize their virtual money. To achieve this, the SG establishes a pre-determined scenario through which participants must progress for 20 min. This time can be extended by collecting watches scattered throughout the scenario or by purchasing them from vending machines in exchange for a portion of the accumulated money. Along the journey, money can be earned through certain tokens (see Table 1 for a detailed list). Some, such as coins, offer guaranteed gains simply by collecting them. Others, like boxes, may offer gains if you decide to open them, but they can also lead to losses. Risk boxes indicate the potential outcomes as well as their probability (risk contexts); while ambiguous boxes provide no information (ambiguous contexts), thus offering a range of decision-making from complete certainty to complete uncertainty.

**Table 1:** Tokens that can be found during KDT and the different variables that are registered based on these.

	Token	Token description	Variable	Variable description	Dimension
			Total gains	Number of times someone wins during KDT	Performance
			Total losses	Number of times someone losses during KDT	Performance
	Coins	Coins scattered along KDT. Provide certain gains (from $10 to $1000) just by collecting them	Coins collected	Number of coins collected during KDT	Risk behavior
	Mixed coins (MC)	Intermittent coins scattered along KDT. They bring gains if picked up on green, but bring losses if picked up on red. The color changes every second	MC collected	Number of mixed coins collected during KDT	Risk behavior
			MC wins	Number of mixed coins collected in green (gains)	Performance
			MC losses	Number of mixed coins collected in red (losses)	Performance
	Ambiguous boxes (AB)	Boxes that, if opened, can bring gains or losses. The amount at stake and the probability of occurrence is uncertain	AB touched	Number of ambiguous boxes touched	Risk behavior
			AB accepted	Number of ambiguous boxes opened	Risk behavior
	Risk boxes (RB)	Boxes that, if opened, can bring gains or losses. The amount at stake and the probability of occurrence is known	RB touched	Number of risk boxes touched	Risk behavior
			RB accepted	Number of risk boxes opened	Risk behavior
	Banks	Cash machines spread along KDT where you can deposit all the money earned in exchange for a 50% commission	Bank entrances	Number of times someone enters the bank	Risk behavior
			Bank deposits	Number of times someone deposits money in the bank	Risk behavior
	Watches	Watches scattered along KDT. Provide a certain time increase by simply picking them up	Watches purchased	Number of watches purchased	Risk behavior
			Watches collected	Number of watches collected for free during KDT	Risk behavior
	Shields	Shields scattered along KDT. Once collected, it can be activated at any time and lasts for 10 s. During this time, no danger can harm you, but neither can money be collected, nor boxes be opened	Shields purchased	Number of shields purchased	Risk behavior
			Shields collected	Number of shields collected for free during KDT	Risk behavior
			Shields activated	Number of shields activated	Risk behavior
	Dangers	Robots, spikes, mines, and fire scattered throughout KDT and whose contact causes economic losses	Dangers suffered	Number of damages suffered during KDT	Performance

Additionally, the distribution of these tokens follows a specific logic: the KDT scenario features branching paths or entrances to various rooms, requiring participants to implicitly choose which way to advance. Although participants may not be aware of it, these choices always involve choosing between safe zones or paths (grey floor) and dangerous zones or paths (red floor). In both cases, participants may encounter the aforementioned tokens; however, in the hazardous regions, coins are guarded by enemies or traps (refer to Table 1 for a detailed list) that can cause them to lose more money than they could gain, and in these areas, boxes (both risk and ambiguity) are preconfigured for participants to lose. In safe regions, on the other hand, coins are easy to obtain; they may still be guarded by certain dangers, so there is some risk involved, but it is easy to obtain them, and it compensates for the amount of profits that can be gained compared to potential losses. Moreover, although the boxes carry some probability of loss, they generally lead to gains if participants choose to open them in these secure contexts. Thus, the most adaptive decision-making strategy in KDT is to take the risk of collecting coins and opening all boxes, but only in safe regions. As we can see, this entails taking risks, but not indiscriminately; risks should only be assumed in a specific, safe context. This difference between dangerous and safe areas is not explicit, being necessary to learn through reinforcement that it is not advisable to enter regions with a red floor, nor to open boxes in these areas. This configuration was chosen to emulate the complexity of many real-life decisions where information is not explicit and must be learned from past experience. Thus, although KDT poses several simpler isolated decisional contexts, in general terms, participants are expected to start by exploring the whole environment and, progressively, after feedback, learn to choose the safest areas.

It should also be noted that at some points along the journey, or by purchasing them from vending machines, shields can be found. If activated, shields protect against money losses for 10 s, allowing participants to exit dangerous regions without incurring damage, but no earnings can be obtained during that time. Additionally, ATMs where accumulated money can be stored to prevent loss can also be found, but always at the cost of a steep fee. Finally, if participants reach the end of the KDT course, they can continue exploring until their remaining time runs out, or they can choose to end the game with the money they have collected so far.

Before undertaking the KDT, participants watched a video tutorial and underwent a 5-min practice session where they were asked to practice navigating the environment and interacting with each token. The serious game runs on a conventional PC and does not require a high-performance graphics card hardware to operate. Participants interacted KDT onto a Screen of 37″ at a distance of 1 m from the participant and with the lights in the room turned off. Moving through the environment required only one hand to control the directional keys on the keyboard, and when necessary, the same hand was used to press 1 (open box) or 2 (do not open), or the space bar to activate the shield.

KDT constitutes a stealth assessment and can provide multiple outcomes or raw data. From each token, many variables could be derived. For instance, from the risk boxes, we could obtain whether participants took the risk to open them or not, but also how much time they took to decide, and how much time they spent based on whether the decision was risky or not. Following the principle of parsimony but avoiding cherry-picking, we first classified these raw data into two theoretical dimensions: on one hand, there are those that only indicate risk-related behaviors (doing something or not doing it, such as buying a shield or opening a box). On the other hand, there are those that indicate performance or consequences of those behaviors, such as total gains or damages suffered. Then, a Principal Component Analysis (PCA) was carried for each block. These analyses demonstrated how variables clustered into logical dimensions. For example, behaviors related to the “boxes” tokens were largely found within a same dimension. This allowed us to identify the variables that were most significant within each dimension and those that were irrelevant or redundant. In the “boxes” dimension, for instance, touching and opening risk boxes, as well as touching and opening ambiguous boxes, were the most influential variables. We could have considered extracting a common factor from this data to treat the “boxes” dimension as a singular variable, but risk boxes and ambiguous boxes, though similar, might represent qualitatively distinct decision-making contexts. Additionally, there is a qualitative difference between merely touching a box and making the decision to open it. To capture the intricacies of the decision-making process, we selected the most representative variables for each dimension as identified by the PCA analysis, avoiding oversimplification.

Finally, a total of 18 variables were selected, categorized within the two theoretical blocks. Five of them belong to performance, including, for example, the total number of gains or losses obtained during KDT. The other 13 are part of the “risk behavior” dimension and encompass behaviors such as opening boxes, depositing money in the bank, or purchasing shields. For a detailed list of these variables and their descriptions, it is recommended to refer to Table 1.

After the extraction of these 18 variables, it was verified whether they were related to classical measures of decision-making, revealing if KDT is a useful tool for evaluating this cognitive ability. Furthermore, hierarchical clustering analysis was used to investigate whether participants clustered into different profiles based on their scores on these variables, revealing different ways of performing or behaving on KDT. This allowed an examination of whether the different profiles exhibited differences on classical measures of decision-making, thereby enhancing the validation of KDT.

### User satisfaction evaluation questionnaire (USEQ)

The USEQ^45^ is a questionnaire designed to properly evaluate the satisfaction of the user (which is part of usability) in virtual systems. It is composed of six items on a 5-point Likert scale, that must be aggregated to obtain a total score. Scores can be interpreted as follows: 6 to 11, low satisfaction; 12 to 17, moderate satisfaction, 18 to 23, good satisfaction; and 24 to 30, excellent satisfaction.

### Mixed gamble task (MGT)

To evaluate decisions in risk contexts, participants performed a short version of MGT^49^. Each trial consisted of a bet with one combination randomly extracted from an 8 × 8 losses and gains matrix, until the 64 combinations were completed. As in the original task^27,49^, gains could range from €100 to €380 in increments of €40, and losses from €50 to €190 in increments of €20. In each trial there was a 50% chance of gaining and losing. Participants had to decide whether to accept or decline the bet. They were instructed that €200 was their initial amount and each bet had to be done with that reference. The Prospect-Theory computational model^50^ was used to analyze decision-making.

Following the original paper of Sokol-Hessner et al.^50^, the utility for gains was estimated through the equation *u(x*^*gain*^*)* = *x*^*ρ*^, and the utility for losses through the equation *u(x*^*loss*^*)* =  *− λ* × *(− x)*^*ρ*^. Finally, the probability of accepting a gamble was estimated using the SoftMax function, P_(Accept)_ = 1/(1 + e^−μ(U(Accept)−U(Reject))^). The model produces three parameters: *λ* (loss aversion), *ρ* (risk attitude), and *μ* (consistency). A value of *λ* = 1 indicates that gains and losses were valued equally, while *λ* > 1 indicated overvaluation of relative to gains (loss aversion). A smaller *ρ* represents a higher risk aversion, relative to a larger *ρ*. A *ρ* value of one indicates risk neutrality. *μ* represents the amount of “randomness” in the subject’s choices or, in other words, consistency over choices. Higher levels of the parameter would indicate that participants rely more on rule-based decision-making^50^.

The parameters for each participant were estimated using Hierarchical Bayesian Analyses^51^, performed with the hBayesDM package^52^ for the R software. The hBayesDM uses Stan 2.1.1^53^ with the Hamiltonian Monte Carlo (HMC) algorithm as MCMC for sampling the posterior distributions. Following Alacreu-Crespo et al.^54^, we drawn 40.000 samples, after burn-in of 23.333 samples, in three different chains (in sum, a total of 120.000 samples and 70.000 burn-in). We used the Gelman-Rubin test^55^ to study if the chains converged (*Ȓ*) to the target distribution. *Ȓ* values were 1, which means that convergence was achieved. In addition, to confirm this convergence, the MCMC chains were visually inspected.

### Iowa gambling task (IGT)

The computerized version of the IGT^28,29^ was carried out to assess ambiguous decision-making. Participants should get the maximum benefit possible over 100 consecutive decisions where they can win and lose money. They can choose from four decks of cards: two disadvantageous (A and B) and two advantageous (C and D). A and B provide large immediate gains, but large losses in the long run. C and D provide lower short-term gains, but lower long-term losses, so their choice leads to higher profit. After each decision, participant receives feedback that can be used to adjust future decisions. Performance was assessed by calculating the Iowa Gambling (IG) index: selections of C and D minus selections of A and B. The higher the IG index, the better the performance.

### Stimulating & instrumental risk questionnaire (S&IRQ)

The S&IRQ^56^ is composed by 7 items on a 5-point Likert scale that allows the evaluation of the two main motives behind risk taking: pleasure (stimulating risk; 4 items), or achieving an important goal (instrumental risk; 3 items). Stimulating risk score can range from 4 to 20 points, and instrumental risk from 3 to 15. In both cases, the higher the score, the higher the propensity for each type of risk.

### Procedure

This study was approved by the Ethics Research Committee of the University of Valencia in accordance with the ethical standards of the 1969 Declaration of Helsinki. Experimental sessions were conducted between 11:00 am and 7:00 pm and lasted approximately one and a half hours. All participants were cited in the laboratory, signed informed-consent, and were submitted to KDT. Immediately after KDT, videogame’s usability was evaluated through the USEQ, and participants were given traditional assessments of their decision-making skills using the MGT, the IGT and the S&IRQ.

### Statistical analyses

Outliers were analyzed using the 3 standard deviations method. Only the *λ* parameter (loss aversion) showed outliers, with two participants scoring slightly above the cut-off. We followed the recommended treatment for outliers^57^, and performed our analyses with and without these participants to check for significant differences. The results obtained were very similar. On the other hand, the Kolmogorov–Smirnov (K-S) test with Lilliefors correction was employed to assess normality. According to the K-S test, most of the data were normally distributed (*p* > 0.05). Exceptions were the scores on the IGT and the instrumental risk dimension of the S&RQ, which deviated significantly from normality. However, as Field^57^ points out, considering the restrictive nature of the K-S test, the Q-Q plot for these variables was also examined. This analysis revealed that their distributions were in fact very close to normality, allowing the use of parametric tests. Analyses included Pearson’s correlations between KDT outcomes and classical decision-making measures (from MGT, IGT and S&IRQ). Moreover, hierarchical clustering was utilized to classify participants into different groups according to their KDT performance. Then, differences between groups in classical decision-making measures were tested through MANOVA. All analyses were controlled for gender and videogame ability level. The α significance level was set at 0.05 and partial eta square (η^2^_p_) symbolizes the effect size. All analyses were performed with IBM SPSS Statistics 25, excepting for the hierarchical clustering that was carried out with Orange 3.35.

## Results

### Sample description

First, to put the following results into context, a sociodemographic description of the sample is shown below. All participants were healthy and young people (age: *M* = 19.73, *SD* = 2.72), from Spain, with a BMI (*M* = 21.77, *SD* = 2.34) within the normal range (18.5–24.9), and with an intermediate socio-economic status (*M* = 3.17, SD = 0.58), assessed by an ad hoc Likert-type question with 5 points, where 1 represents the lowest status and 5 the highest. Furthermore, all of them were undergraduate or master’s degree university students in health sciences, and 14 out of the 81 participants were juggling these studies with their jobs.

### Usability

The average USEQ score of our sample was 24.99 (*SD* = 3.19) out of 30, which indicated that user satisfaction using KDT was excellent, in line of our first hypothesis. A detailed analysis of the items revealed that 90.1% of the participants had quite a lot or a lot of fun using KDT. 96.3% felt that the information on KDT was quite clear or very clear. 63% performed fairly or very satisfactorily and 61.7% felt that they had a lot or a fair amount of control over KDT. Finally, 71.6% felt little or no discomfort and 88.6% indicated that they felt little or no motion sickness while playing.

### Relationship between the KDT outcomes and classical decision-making measures

To test the second hypothesis of our study, Pearson’s correlations were performed in order to examine the association between KDT scores and classical decision-making measures (MGT, IGT, and S&IRQ). Several significant relationships were found (see Table 2).

**Table 2:** Pearson’s correlations between KDT scores on the variables described in Table 1 and the classical decision-making measures obtained by MGT, IGT and S&IRQ.

		MGT	MGT	MGT	IGT	S&IRQ	S&IRQ
		Risk attitude	Loss aversion	Consistency	IG index	Stimulating risk	Instrumental risk
KDT outcomes	Total gains	0.337**	− 0.048	0.297**	− 0.038	0.151	− 0.223*
	Total losses	− 0.040	− 0.122	− 0.064	− 0.259*	0.350**	− 0.211
	Coins collected	0.320**	− 0.010	0.294**	− 0.003	0.085	− 0.238*
	Mixed Coins collected	0.171	0.035	0.174	− 0.039	0.057	− 0.038
	Mixed Coins wins	0.184	0.064	0.211	− 0.037	0.065	− 0.030
	Mixed Coins losses	0.037	− 0.064	− 0.026	− 0.020	0.004	− 0.037
	Ambiguous boxes touched	− 0.009	− 0.186	− 0.096	− 0.135	0.237*	− 0.216
	Ambiguous boxes accepted	0.074	− 0.204	− 0.021	− 0.104	0.281*	− 0.178
	Risk boxes touched	0.033	− 0.298**	− 0.006	− 0.229*	0.093	− 0.097
	Risk boxes accepted	0.154	− 0.316**	0.082	− 0.219*	0.280*	− 0.202
	Bank entrances	0.354**	0.114	0.360**	0.083	− 0.186	− 0.083
	Bank deposits	− 0.252*	0.150	− 0.157	0.090	− 0.001	− 0.060
	Watches collected	0.046	− 0.100	0.012	− 0.054	− 0.024	− 0.061
	Watches purchased	0.132	0.063	0.125	0.272*	0.037	− 0.034
	Shields collected	− 0.100	− 0.039	− 0.113	− 0.088	0.132	0.191
	Shields purchased	0.035	− 0.019	0.019	0.175	− 0.107	0.006
	Shields activated	0.027	− 0.067	− 0.023	0.167	− 0.028	0.135
	Dangers suffered	− 0.076	− 0.055	− 0.067	− 0.245*	0.305**	− 0.172

*p* < .05; *p* < .01.

Starting with the risk decision-making contexts, as evaluated by the MGT, the three parameters derived from this task showed notable associations with KDT. Firstly, the risk attitude parameter (*ρ*) exhibited a positive correlation with the number of gains achieved in KDT, the quantity of collected coins, and entries into the bank, but a negative correlation with the deposits made in it. Additionally, the greater the loss aversion (*λ*), the fewer times participants touched and opened the risk boxes. Finally, greater consistency (*μ*) in MGT decisions corresponded to a higher number of gains, coins collected, and bank entries observed in KDT. Furthermore, with regard to decision-making in ambiguous contexts, as evaluated by the IGT, it was observed that better performance in making decisions in this task (i.e., a higher overall score on the IGT) was negatively associated with the number of losses and overall damages suffered, as well as the number of risk boxes touched and opened in KDT. Moreover, a higher IG index correlated with a greater number of watches purchased by the participants. Lastly, the self-reports obtained with the S&IRQ were analyzed with regard to the motives that drive the participants to take risks. Those who scored higher in the pursuit of stimulating risks also touched and opened more ambiguous boxes and opened more risk boxes in KDT. Additionally, they also experienced more losses and overall damages. On the other hand, those who scored higher on instrumental risk, while not appearing to suffer more damages or losses, collected fewer coins, and won less frequently.

### Clusters extracted from KDT outcomes

In addition to the correlations obtained, hierarchical clustering was conducted to test the third hypothesis, which involves classifying participants into different groups based on their similar performance in KDT. Ward’s Linkage was utilized as a way of conceptualizing the locations of clusters, and Squared Euclidean Distance as a way of measuring the distances between cases and clusters. This analysis suggested a three-cluster solution, which was also confirmed through K-means clustering by obtaining this solution the higher silhouette score.

To understand the theoretical meaning of each cluster, a MANOVA was conducted to compare KDT scores across clusters. Cluster 1 appeared to group the most risk-avoidant participants, who took less risk in exchange for not making too much money. Clusters 2 and 3, on the other hand, grouped participants who took more risks than Cluster 1. However, Cluster 2 took more useless and impulsive risks, making much more losses; contrary to Cluster 3, which only took relatively safe and useful risks, which resulted in participants winning and not losing too much money. Specifically, as can be seen in Table 3, Risk-avoidant group (Cluster 1) and Useful risk group (Cluster 3) touched and opened a similar number of both ambiguous and risk boxes. However, the Useful risk group (Cluster 3) risked more when collecting coins and mixed coins than the Risk-avoidant group (Cluster 1), which also led them to achieve a higher number of mixed coins wins and total gains. By the other side, the Useless risk group (Cluster 2) collected a similar number of coins and mixed coins than the Useful risk group (Cluster 3), as well as gained a similar mixed coins wins and total gains, but also touched and opened more ambiguous and risk boxes, suffered more damages from dangers and accumulated a higher total losses than the Risk-avoidant (Clusters 1) and Useful risk (Cluster 3) groups.

**Table 3:** Differences between the three clusters in the scores obtained on the KDT outcomes: Cluster 1 or Risk-avoidant group; Cluster 2 or Useless risk group; and Cluster 3 or Useful risk group.

		Cluster 1 Risk-avoidant group (*N* = 39)	Cluster 2 Useless risk group (*N* = 21)	Cluster 3 Useful risk group (*N* = 21)	*F*	df between	df within	*p*	η^2^_p_
KDT outcomes	Total gains	^a^140.03 ± 16.17	^b^163.24 ± 12.67	^b^162.9 ± 9.46	28.02	2	78	< 0.0001	0.41
	Total losses	^a^30.92 ± 8.68	^b^47.05 ± 14.28	^a^30.67 ± 6.63	20.38	2	78	< 0.0001	0.34
	Coins collected	^a^127.31 ± 14.09	^b^137.71 ± 9.84	^b^138.43 ± 5.32	9.11	2	78	< 0.0001	0.19
	Mixed Coins collected	^a^8.51 ± 4.14	^b^16.86 ± 8.97	^b^19.52 ± 9.72	18.47	2	78	< 0.0001	0.32
	Mixed Coins wins	^a^4.23 ± 3.46	^b^10.33 ± 6.76	^b^14.05 ± 9.42	17.68	2	78	< 0.0001	0.31
	Mixed Coins losses	^a^4.28 ± 1.74	^b^6.52 ± 4.07	^ab^5.48 ± 2.35	4.97	2	78	0.009	0.11
	Ambiguous boxes touched	^a^13.59 ± 5.45	^b^25 ± 4.89	^a^16.62 ± 6.61	28.14	2	78	< 0.0001	0.42
	Ambiguous boxes accepted	^a^9.64 ± 5.56	^b^20.76 ± 5.33	^a^13.14 ± 5.67	27.66	2	78	< 0.0001	0.42
	Risk boxes touched	^a^6.15 ± 1.89	^b^9 ± 2.4	^a^5.62 ± 1.74	18.26	2	78	< 0.001	0.32
	Risk boxes accepted	^a^ 3.44 ± 1.63	^b^5.43 ± 1.43	^a^4.24 ± 1.54	11.12	2	78	< 0.001	0.22
	Bank entrances	^a^3.38 ± 0.93	^a^3.1 ± 1.22	^a^3.71 ± 0.9	1.98	2	78	0.145	0.05
	Bank deposits	^a^1.18 ± 1.25	^a^0.57 ± 0.74	^a^0.62 ± 0.74	3.31	2	78	0.042	0.08
	Watches collected	^a^1.74 ± 0.78	^a^2.1 ± 0.7	^a^1.95 ± 0.59	1.74	2	78	0.181	0.04
	Watches purchased	^a^0.33 ± 0.62	^a^0.9 ± 1.04	^b^2.19 ± 2.06	15.12	2	78	< 0.001	0.28
	Shields collected	^a^0.82 ± 0.38	^a^1 ± 0.1	^b^0.29 ± 0.46	23.31	2	78	< 0.001	0.37
	Shields purchased	^a^0.51 ± 0.68	^ab^0.62 ± 0.74	^b^1.1 ± 0.62	5.11	2	78	0.008	0.12
	Shields activated	^a^1.26 ± 0.63	^a^1.62 ± 0.74	^a^1.38 ± 0.59	2.1	2	78	0.129	0.05
	Dangers suffered	^a^22.05 ± 7.85	^b^29.52 ± 12.15	^a^18.23 ± 5.64	9.23	2	78	< 0.001	0.19

Mean ± SD (means with a different super index ^a^ or ^b^ indicate that Bonferroni post hoc comparisons between clusters revealed significant differences in that KDT variable); df, degrees of freedom.

### Differences between clusters in classical decision-making measures

**Figure 2:**
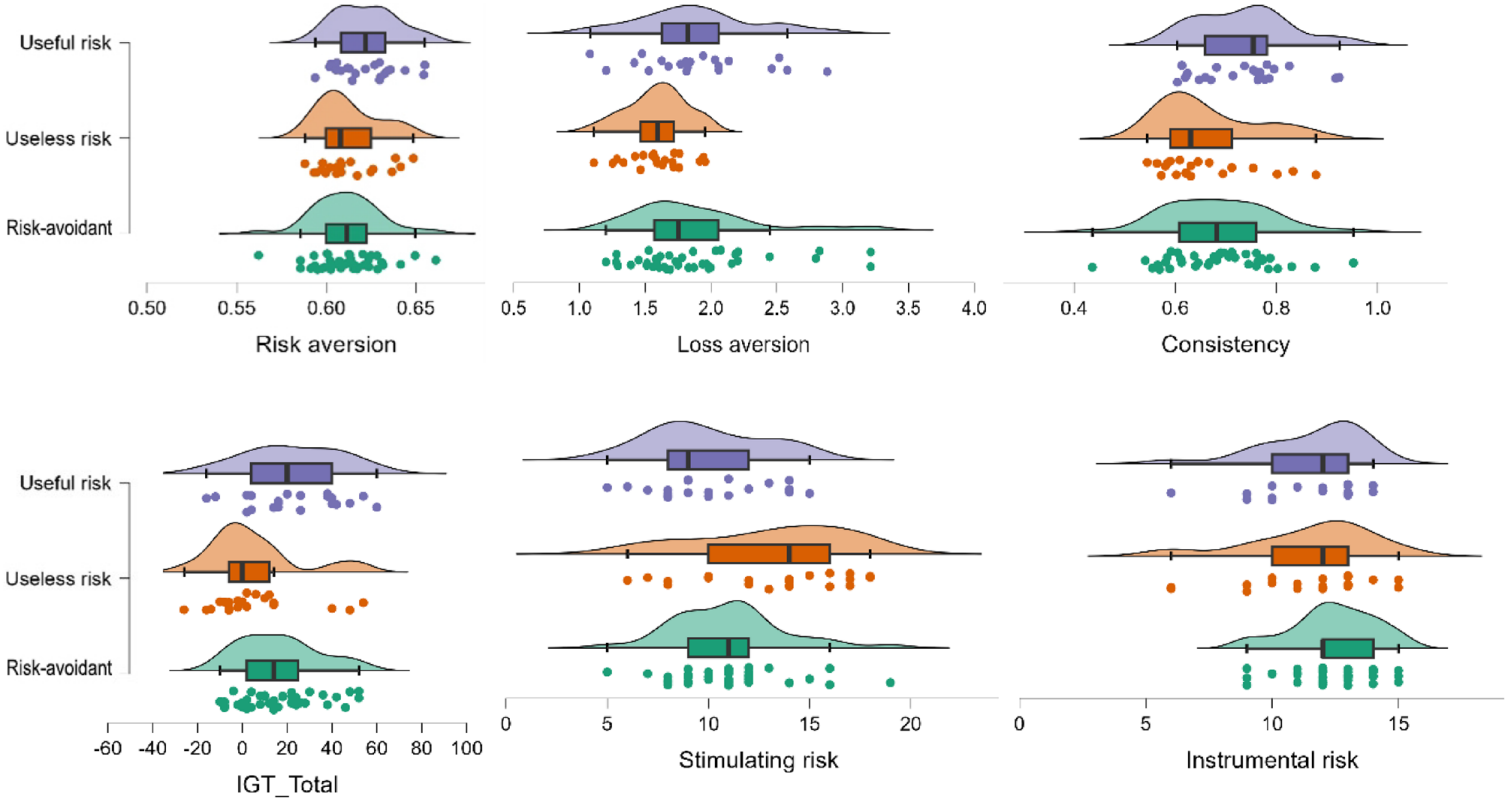
Raincloud plots (jittered individual points, box plot and halved violin plot) according to cluster membership for risk attitude, loss aversion, decisions consistency, IGT total index, stimulating risk and instrumental risk.

Finally, with regard to the fourth hypothesis, a MANOVA was conducted to ascertain the existence of differences in classical measures of decision-making between the three clusters classified by KDT scores. As can be seen in Table 3 and Fig. 2, the Useless risk group (Cluster 2) showed significantly lower loss aversion in MGT, and higher scores in stimulant risk assessed with S&IRQ, than Risk-avoidant (Cluster 1) and Useful risk (Cluster 3) groups; additionally, the Useless risk group also scored significantly lower than the Useful risk group on the IGT and showed a significant trend (*p* = 0.070) towards less consistency in their decisions as assessed by the MGT than the latter group (Table 4).

**Table 4:** Differences between clusters in classical decision-making measures (MGT, IGT, S&IRQ tests).

	Cluster 1 Risk-avoidant group (*N* = 39)	Cluster 2 Useless risk group (*N* = 21)	Cluster 3 Useful risk group (*N* = 21)	*F*	df between	df within	*p*	η^2^_p_
MGT risk attitude	^a^0.61 ± 0.018	^a^0.61 ± 0.017	^a^0.62 ± 0.017	2.71	2	78	0.072	0.06
MGT loss aversion	^a^1.87 ± 0.48	^b^1.59 ± 0.22	^a^1.89 ± 0.45	3.61	2	78	0.032	0.08
MGT consistency	^a^0.68 ± 0.1	^a^0.66 ± 0.09	^a^0.73 ± 0.09	2.99	2	78	0.05	0.07
IG index	^ab^16.26 ± 17.46	^a^5.05 ± 20.46	^a^22.76 ± 21.15	4.59	2	78	0.013	0.11
S&IRQ stimulating risk	^a^11 ± 2.71	^b^13.14 ± 3.73	^a^9.95 ± 2.83	6.12	2	78	0.003	0.14
S&IRQ instrumental risk	^a^12.44 ± 1.65	^a^11.52 ± 2.48	^a^11.62 ± 2.01	1.92	2	78	0.152	0.04

Mean ± SD (means with a different super index ^a^ or ^b^ indicate that Bonferroni *post hoc* comparisons between clusters revealed significant differences in that KDT variable); df, degrees of freedom

## Discussion

The aims of this study were to introduce, test the usability, and validate Kalliste Decision Task (KDT), a serious game based in a virtual environment designed for a comprehensive and ecological assessment of decision-making assessment. As hypothesized, the USEQ scores showed that user satisfaction was excellent, revealing the high usability of KDT. Moreover, in line with our second hypothesis, the majority of KDT behaviors were related to multiple classical decision-making variables, allowing the prediction of how participants would decide in both risky and ambiguous contexts based on their KDT performance. These results demonstrate that KDT is a valuable tool in the field of decision-making research, allowing for comprehensive assessments at a low cost and effort. They are discussed in more detail below.

Decision-making in risk contexts is usually assessed by the MGT^27^, which provides three parameters^50^, that have been related to the variables obtained in KDT. In our study, risk attitude (*ρ*) was positively correlated with the number of coins collected and the total gains in KDT. Since a higher *ρ* represents a lower risk aversion^50^, this means that the lower the risk aversion in MGT, the greater the approximation to the coins and, therefore, the higher the number of gains achieved in KDT. This result is in line with the KDT settings, as exploring and taking some degree of risk would be associated by higher gains than being overly cautious. Additionally, risk attitude was also positively related to the bank entrances, which might seem contradictory. Yet, the lower the risk aversion, the lower number of bank deposits was found, suggesting that these participants did not choose to protect their money in exchange for a commission, taking the risk of potentially losing it. Complementarily, the higher the consistency parameter (*μ*), which represents a higher rule-based or reflective decision-making^50^, the higher number of gains, coins collected and bank entrances. This relationship makes sense, as individuals who make highly rational or rule-guided decisions are typically the same ones who express less risk aversion^50,58,59^, which may avoid extremely cautious behaviors on KDT and encourage the greater acquisition of coins. Nevertheless, such a thoughtful strategy could facilitate the assumption of risks only in those contexts where taking risks is advantageous, i.e., in safe zones. Moreover, entering the bank more often, regardless of whether they deposit the money or not, could be considered as a reflective behavior. Finally, loss aversion (*λ*), the most important parameter in a risky decisional context such as MGT^27^, was negatively correlated with the number of risk boxes touched and, especially, accepted. In accordance with our second hypothesis, this result is logical as the risk boxes resemble the format of the MGT^27^, which specifies the potential gains and losses, as well as the probabilities of their occurrence (i.e., risk boxes also constitute a risk context). Thus, just as lower bet acceptance indicates greater loss aversion in the MGT, lower opening of the risk boxes would indicate greater loss aversion in KDT.

By the other side, ambiguous decision-making use to be evaluated through the IGT^28,29^. There is a debate about whether all IGT is ambiguous, or only the first 40 decisions are. This is because as participants gain knowledge of the task, it might more closely resemble a context of risk^60^. However, the point at which the task transitions from ambiguous to risky can vary depending on multiple factors^61^, and because information about possible outcomes and their probabilities is never provided at any point in the task (as is the case in risk tasks), much of the literature continues to treat the complete IGT as an ambiguous decision-making context^7,62^, which is the approach we have adopted in our work.

A higher IG index would represent a better decision-making performance, characterized by the ability to delay immediate gains in exchange for avoiding large losses and obtaining large, delayed gains. Continuing with the second hypothesis, our results revealed that the higher the IG index, the lower the damage suffered, and the lower the losses obtained in KDT. In our virtual environment, many gains are located in very dangerous zones that may entail significant big losses. Thus, this result would indicate that those people who prefer to delay rewards by avoiding large losses in IGT would be the same people who learn to avoid the dangers and have fewer total losses in KDT, risking only in the safe zones. Furthermore, the higher the IG index, the lower the number of risk boxes touched and opened. As previous literature proposed, an important component underlying the decisional process in IGT is also loss aversion^51,52^. It has been highlighted that loss aversion could facilitate the reinforcement-learning process in IGT by increasing punishments sensitivity^26,63^. Since risk boxes acceptances were correlated with loss aversion in MGT, it could be possible that they were also correlated with loss aversion in IGT. To confirm this, future research should build on new developments in computational modelling^52,62^ that allow the extraction of sub-components such as loss aversion in complex tasks such as IGT. These specific approaches should also address the cognitive mechanisms underlying the ambiguous boxes in KDT in order to elucidate the absence of a relationship found between them and the IGT performance. Despite the hypothesis that these boxes, as they represent a context of high uncertainty (ambiguous context), should correlate with IGT performance, no such relationship was observed.

To conclude our approach to the second hypothesis, we assessed risk taking through the S&IRQ^56^. Those people who scored high on instrumental risk, i.e., those who only take risk when it is necessary to achieve a goal, also collected lesser coins and won fewer times in KDT. This relationship also makes sense because many coins in KDT are in completely safe and easily accessible places, which could lead participants who obtain them to believe that they are achieving the goal set in KDT, which is to earn the maximum amount of money. However, as mentioned above, it is necessary to take certain risks in safe zones in order to maximize the acquisition of coins and profits, facing some dangers that are easily avoidable and entail few losses. Conversely, as expected, those who enjoy with stimulant risks were the same ones who opened a greater number of both risky and ambiguous boxes, as well as those who suffered more damages and accumulated more losses. This seems to be a clear indicator that they were willing to take risks even when they were not necessary, i.e., regardless of whether they were in safe or dangerous areas, just for fun. Although self-reports could present important differences with tasks such as MGT or IGT^37^, KDT also provided important relationships with the S&IRQ, indicating that our tool was also able to bring information also on more cold or dispositional, and not only hot or situational measures.

As hypothesized, multiple KDT tokens have been linked to a range of classical measures of decision-making. To date, as previously indicated, other SG such as Spheres & Shields Maze Task had been developed^43^, which significantly predicted risk-related factors such as impulsivity, extraversion, or behaviors such as marijuana consumption. However, KDT represents a SG capable of providing direct insights into how individuals make decisions in different decision contexts, ranging from low to high ambiguity, both situationally and dispositionally, all within a single assessment. Although many of the correlations found were moderate or weak and could disappear with a correction for multiple comparisons, these analyses served an exploratory purpose by identifying which KDT variables have the greater potential to inform decision-making. As the literature suggests^64,65^, such unguided analyses benefit from not applying corrections, prioritizing the reduction of Type II over Type I error. However, a new phase of validation is now required, with a larger and more diverse sample conducting confirmatory analyses to verify if these preliminary results hold true. Additionally, these new studies should use the information now available to refine KDT. Based on the non-significant correlations found, it should be considered whether some tokens, like mixed coins, should be removed from the tool due to their poor predictive power. Conversely, it should also be evaluated whether these tokens could predict other related variables. For example, mixed coins may not provide information about loss aversion or decision consistency, but due to their intermittent nature, they could offer insights into motor or planning impulsivity. These hypotheses should be tested in future validation phases to make KDT an even more useful tool.

Nevertheless, and beyond the correlation analyses, hierarchical cluster analyses were also conducted to further validate and complement the assessment of KDT. As we anticipated in our third hypothesis, these analyses revealed that participants could be classified into three distinct and theoretically meaningful groups based on their KDT decision-making behavior. Cluster 1 included extremely risk-avoidant participants, who touched and opened a relatively small number of risky and ambiguous boxes and did not risk taking normal and mixed coins. This way of deciding involved less risk, but also less profit, because as noted above, KDT is set up so that a certain degree of risk is rewarded, specifically when it is taken in safe zones. Participants in Cluster 3, in contrast, touched and opened a similar number of risk and ambiguous boxes as those in Cluster 1, but they also risked taking more coins efficiently, making more gains but without increasing damages or losses. Therefore, Cluster 3 therefore represents people who took useful risks. Finally, Cluster 2 also assumed more risks than Cluster 1, but in a useless way. In this case, they also took more coins and increased profits, as well as touched and opened significantly more risky and ambiguous boxes, but regardless of whether they were in a safe or in a dangerous zone; thus, they also suffered more damage and total losses. Since KDT is set up to penalise excessive and unmeasured risk (especially when it is taken in dangerous zones), Cluster 2 would represent participants who took useless risks.

Finally, as we noted with our fourth hypothesis, cluster membership explained a significant percentage of the variance in most of the classical measures of decision-making. The Useful risk group (Cluster 3) and the Risk-avoidant group (Cluster 1) did not differ greatly on the classical measures, although the IGT scores of the useful risk group or the consistency of their decisions on MGT were slightly higher than those of the Risk-avoidant group, whose scores were closer to the Useless risk group (Cluster 2). In contrast, the Useless risk group clearly showed the highest preference for stimulating risks. Moreover, they showed the lowest loss aversion on the MGT, which may indicate more rational decisions in risk contexts^9,15,66^, but also the lowest IG index, indicating that they made poor decisions in more complex or ambiguous contexts such as IGT^13,26^ or the own KDT.

These findings are consistent with previous literature highlighting the importance of emotions, and specifically loss aversion, when making decisions. As introduced, patients with emotional-brain injuries maintained an intact IQ but did not express loss aversion and had hard difficulties to decide in complex contexts since they have significant difficulties in grasping feedback following their decisions and learning through reinforcement^29,67^. In this line, several patients such as suicide attempters, expressed lower loss aversion and worse IGT performance than healthy controls^23,24^. In this case, we have seen that people who manifest lower loss aversion are also the same ones who take unnecessary risks, experiencing harm and losing more money, without learning that the most advantageous way to achieve greater benefits in KDT is to adopt a more balanced strategy. Now, it remains to be verified, through future studies, whether these participants categorized as engaging in useless risk in KDT also exhibit other characteristics that may be associated with this decision-making profile. This could include poorer emotional regulation or identification^68,69^, higher levels of stress^7,70^, or even reduced availability of dopamine in their reward centers^71–73^. Additionally, it would be essential to use KDT in clinical populations to assess whether it can detect decision-making deficits specific to different pathologies^74^, thereby revealing differences in gaming patterns compared to the healthy population or potentially creating new clusters that facilitate diagnosis.

The results obtained through hierarchical clustering appear to support what was already observed through correlations. Furthermore, this method offers the advantage of facilitating the identification of each participant’s decision-making pattern based on their membership in one of the three clusters. If these clusters are confirmed in future studies, by increasing the sample size and heterogeneity (including different age groups, educational levels, cultures, clinical diagnoses, etc.), KDT could become a powerful tool for the rapid and effective assessment of decision-making abilities. This could potentially resolve the ongoing debates regarding the validity of classical economic models as predictors and/or descriptors of human decision-making behavior^12,75^ and be in line with the emerging trends in the decision neuroscience^76^. Furthermore, its implementation across various populations, regardless of age or experience with video games, seems feasible due to the ease and user-friendliness revealed by usability analyses. In addition, the adaptability of KDT to other hardware platforms, such as controllers or augmented reality systems, as well as the possibility to use it in parallel with neural or physiological measurements, adds to its versatility.

Despite its potential, the validation of KDT has its limitations, with the generalizability of results being the primary concern at this stage. Firstly, our sample consisted of young Spanish university students, which limits the applicability of our findings to the general population. Furthermore, although our sample size was similar to those used in validations of similar tools, it was relatively small. This may raise doubts on the validity of certain analyses, such as cluster extraction, which typically requires larger samples to ensure stability. Future validation phases would benefit from a larger and more heterogeneous sample. This would allow, for example, part of the sample to be used as an independent dataset to confirm the extracted clusters or even to conduct more advanced analyses, such as machine learning predictive models^44,77^. However, due to the exploratory and preliminary nature of our study, corrections for multiple comparisons were not applied, which could have increased the Type I error, questioning the reliability of the results. Therefore, in the next phases of validation, specific confirmatory analyses that verify our propositions should be conducted. Similarly, while KDT offers a stealth assessment that could provide greater ecological potential, its predictive capacity for real-world decisions must be confirmed through direct comparison to real-world setting. Lastly, although participants were motivated after KDT, fatigue and boredom could have affected their performance in subsequent tasks due to the length of the protocol. This suggests that future protocols should be as brief as possible to ensure participants remain engaged.

## Conclusions

Although further research is needed to confirm the utility of KDT, this work establishes a solid foundation. Thus, KDT fulfilled its goal of providing a comprehensive assessment of decision-making. By playing KDT for only 20 min, many of the results obtained were related to and provided information on classical decision-making measures, in both risk and ambiguous contexts, as well as in self-reports. This makes KDT a very useful tool not only for advancing the field of decision-making but also as an ecological and efficient assessment tool that, if validated, could also be valuable even for clinical diagnosis. In this regard, the potential of KDT would extend beyond its current applications, offering promising avenues for integration into clinical workflows. Its efficiency and ecological validity suggest that KDT could serve as a cornerstone in the development of more nuanced, patient-centered approaches to diagnosis and understanding of decision-making processes. Further research could explore its utility in diverse clinical settings, potentially offering a breakthrough in how cognitive assessments are conducted and interpreted in the context of real-world decision-making challenges.

## Data Availability

The dataset generated and analyzed during the current study is available from the corresponding author on reasonable request.
